# The Psychological Impact of Anterior Teeth Caries and Treatment Needs Among Adults at King Saud University Clinics in Riyadh, Saudi Arabia

**DOI:** 10.7759/cureus.68115

**Published:** 2024-08-29

**Authors:** Dalal Aldabeeb, Adeem Alofi, Reem Alfaran, Alanoud Salam, Lujain Alsuhaibani, Maram Alanazi

**Affiliations:** 1 Department of Restorative Dental Sciences, King Saud University, Riyadh, SAU; 2 Department of Preventive Dental Sciences, King Saud Bin Abdulaziz University for Health Sciences, Riyadh, SAU; 3 Department of Public Health, King Abdullah International Medical Research Center, Riyadh, SAU; 4 College of Dentistry, King Saud University, Riyadh, SAU

**Keywords:** anterior teeth, emotional, restorative, psychological, caries, aesthetic

## Abstract

Introduction

Aesthetic dentistry has become increasingly important, as a smile is key to social and emotional well-being. Dental caries can greatly and persistently diminish overall oral health-related quality of life, impacting one's capacity to engage socially and maintain emotional health. The study aims to assess the psychological impact of anterior teeth caries and restorative treatment needs.

Methodology

A cross-sectional study was conducted at King Saud University clinics. The questionnaire used in this study was developed based on previous research and structured into three main domains: demographic data, psychosocial impact of dental aesthetics, and restorative treatment needs assessment. The statistical analysis of the study's Inter-rater reliability was assessed using the Fleiss kappa coefficient. The intra-rater reliability was evaluated for all raters using Cohen's kappa, with each rater showing almost perfect agreement in dental caries and treatment option measures (p < 0.001).

Results

The participants who perceived the presence of anterior caries had significantly worse overall psychological impact scores than those who did not perceive having anterior caries. The overall Psychosocial Impact of the Dental Aesthetics Questionnaire (PIDAQ) score shows a more negative psychosocial impact of dental aesthetics on those needing indirect restoration than those needing direct restorations.

Conclusions

Anterior teeth caries significantly impacts individuals' psychological well-being, particularly in emotional and social aspects. Those perceiving the presence of anterior caries experience more significant emotional distress and social implications. The need for restorative treatment, especially indirect restoration, is associated with higher psychosocial impact and lower dental self-confidence.

## Introduction

Aesthetic dentistry has recently gained importance as a smile is crucial to one's social and emotional well-being. In our beauty-conscious society, a person's smile acts as a window, influencing how they interact with others. Consequently, dental caries significantly and persistently reduces individuals' overall oral health-related quality of life (OHRQOL), significantly influencing their ability to engage in social interactions and maintain emotional well [1-4].

The Global Burden of Disease Study in 2017 identified untreated dental caries as the most prevalent oral health issue worldwide. This condition impacted an estimated 2.3 billion people, representing 32% of the world's population, who had permanent teeth affected by dental caries in 2017. The study showed that dental caries is a significant global public health problem that affects a large number of individuals [5]. The high prevalence of dental caries in Saudi Arabia has been extensively documented in recent years, generating significant public health concerns [6,7].

Also, the 2019 National Oral Health Survey found extremely high rates of dental caries, with 97% of 35-44-year-olds and 91% of 65-74-year-olds affected [8]. An appreciable number of adults reported one form of psychosocial problem because of anterior teeth [1,2].

A study by Khattak et al. revealed a 32.25% prevalence of dental caries in anterior teeth, with the maxillary central incisors identified as being the most affected teeth [9].

Previous studies have identified discrepancies between clinically evident dental problems (normative needs) and individuals' self-perceived needs for dental care. These discrepancies highlighted the importance of measuring people's value to oral health and their behavior in seeking dental treatments [10,11]. 

Self-reported cavities, teeth that look bad, and toothache were strongly associated with self-reported perceived need for dental care [10]. The result of one study conducted on Mexican schoolchildren regarding dental caries self-report indicates the presence of socioeconomic inequalities. Not all the socioeconomic indicators related to dental caries self-report at the ecological level, but regarding poverty and extreme poverty, firmly established evaluations found a strong association [12]. Another study suggested that self-reported dental caries variables at the individual level include older age, low education level, recurrence of dental office visits, and unfulfilled dental needs, all of which must be evaluated carefully [13]. A previous study showed the influence of gender and age on self-perception of oral health, and the likelihood of seeking dental treatments was examined. The study found that women and individuals aged 40 and older experienced a more significant decline in OHRQOL [14]. Patient knowledge about their decayed teeth and their restorative treatment needs assessment helps healthcare authorities to strategically plan dental care services, educational programs, and motivation [11].

Untreated cavities, unattractive or discolored fillings, and missing front teeth commonly lead to dissatisfaction with dental appearance. Treatments that enhance dental aesthetics improve the patient's quality of life and psychological condition. Patients interested in enhancing their dental appearance frequently demand orthodontic treatment, labial veneers, anterior teeth restoration, and tooth whitening treatments [15]. These rely on the dentist's experience and the patient's expectations. The facilities available at dental clinics, the operator's skills, and expertise are critical factors in determining a treatment plan [4]. People usually are unaware of dental diseases until they are diagnosed or treated, though they are aware of the presence of treatment [11].

Research indicates that patients are most likely to report treatment needs related to visible dental problems like remaining roots, mobile teeth, and dental pain. Early-stage dental issues, such as incipient cavities, are less likely to be mentioned [10].

Perceptions of dental appearance vary among individuals and are shaped by cultural and personal preferences. Previous research has noted a notable disparity between how patients and dentists perceive pain and esthetics, particularly concerning color issues [4].

In 2006, Klages et al [16]. introduced the Psychosocial Impact of Dental Aesthetics Questionnaire (PIDAQ), a validated psychometric tool designed to assess how dental aesthetics impact individuals' quality of life, particularly in orthodontic patients. The PIDAQ comprises four subscales and was originally developed in English. When applying this instrument in non-English speaking regions, it necessitates careful translation and cultural adaptation to preserve its psychometric integrity. Numerous translated versions of the PIDAQ have been created and evaluated for reliability and validity, including adaptations for languages such as Arabic [17]. The present study aims to evaluate the psychological impact of anterior teeth caries among adults and assess their restorative treatment needs. This study is designed to assess the hypothesis that anterior teeth caries impact individuals psychologically more than those who do not have anterior teeth caries.

## Materials and methods

This cross-sectional study was conducted at King Saud University clinics in Riyadh, Saudi Arabia, from July 1, 2023, to April 30, 2024.

Participation in this study was voluntary, and a cover letter explaining the research purpose and ensuring data confidentiality was attached to the questionnaire. The study received ethical approval from the Institutional Review Board (IRB) under the reference number E-23-8086 and the College of Dentistry Research Center (CDRC) at King Saud University, Riyadh, Saudi Arabia.

The inclusion criteria for participants were males or females over the age of 18 who were currently receiving dental care at King Saud University clinics and were diagnosed with dental caries (active and/or arrested) on one or more surfaces of their anterior teeth. Exclusion criteria consisted of patients who declined to participate, individuals under the age of 18, any discoloration of the anterior teeth not related to caries, and sound anterior teeth.

The questionnaire used in this study was developed based on previously conducted research [15-17]. It was validated by conducting interviews with 10 randomly selected individuals to assess its clarity, relevance, and simplicity. Necessary modifications were made to ensure the questionnaire's understandability for the target population. The questionnaire is structured into three main domains and included in the appendices.

Domain 1: demographic data 

The first domain focused on collecting demographic data from the participants, including information such as gender, age, educational status, socioeconomic status, and marital status. These demographic factors can provide valuable insights into the study population.

Domain 2: psychosocial impact of dental aesthetics

The second domain of the questionnaire consisted of questions adapted from the PIDAQ, a validated psychometric tool designed to assess how dental aesthetics influence individuals' quality of life. The PIDAQ includes four subscales: A) Dental self-confidence, which evaluates the emotional impact of dental aesthetics with six items; B) Social impact, which examines issues individuals might face in social situations due to perceived unfavorable dental appearance, with eight items; C) Psychological impact, focusing on feelings of inferiority and unhappiness when comparing oneself to others with better dental aesthetics, with six items; and D) Aesthetic concern, assessing disapproval of one's own dental appearance as seen in mirrors, photographs, or videos, with three items. Each item is rated on a 5-point Likert scale from 0 (not at all) to 4 (very strongly), where a score of 0 signifies no impact on the individual's quality of life.

Domain 3: restorative treatment needs assessment

The third domain of the questionnaire included questions that aimed to assess and compare the dentists’ perceptions of restorative treatment needs for each case with the participants’ own perceptions. This domain provides insights into the professional assessment of treatment needs and allows for a comparison with the participants' self-perceived treatment needs.

Participants were seen in the primary care clinics after clinical examinations of anterior teeth using the clinical examination kit, which included a mouth mirror and explorer. The periapical radiographs were used as an examination aid.

All participants gave written informed consent before their participation. The questionnaires were either filled out at the end of the dental visit or sent electronically via their mobile number after the visit. Reminders were sent, and data were collected over five months.

By dividing the questionnaire into these three domains, the study collected data on demographic characteristics, the psychosocial impact of dental aesthetics, and the agreement between dentists' and participants' perceptions of restorative treatment needs. This comprehensive approach allowed for a thorough understanding of the factors that influence individuals' psychological well-being in relation to their caries-affected anterior teeth while also facilitating the assessment of appropriate restorative treatment requirements.

The sample size for this study was suggested to be 185 participants, which was considered statistically significant at a 95% confidence interval and a 7% margin of error.

The statistical analysis of study's inter-rater reliability was assessed using the Fleiss kappa coefficient (used for more than two raters). The overall level of agreement among the four raters when measuring dental caries and deciding on the appropriate treatment option was almost perfect (κ =0.88, p < 0.001) and (κ = 0.91, p < 0.001). The intra-rater reliability was assessed for all four raters using Cohen’s kappa, with each rater showing almost perfect agreement in dental caries and treatment option measures. (p < 0.001).

Participants completed online questionnaires, and the collected data was organized in a password-protected Excel spreadsheet on a secure desktop computer. All data was anonymized using patient file numbers.

## Results

Domain 1: demographic data

Seventy-two patients with anterior teeth caries were examined at the dental clinics at King Saud University. All the participants filled out the questionnaire and were considered the final sample. Most of the participants preferred the Arabic language to complete the questionnaire (N=69, 95.8%), while only 3 out of 72 respondents completed the questionnaire in the English language (N=3, 4.2%). Descriptive statistics of the sample data are shown in Table 1.

**Table 1:** Descriptive statistics and frequencies of the sample variable SAR: Saudi Arabian Riyal

Characteristic	N=72, 100%
Gender:	
Female	N= 65, 90.3%
Male	N=7, 9.7%
Age Range:	
18-23	N=23, 31.9%
24-29	N=12, 16.7%
30-35	N=9, 12.5%
36-41	N=10, 13.9%
42-47	N=7, 9.7%
Older Than 47 Years	N=11, 15.3%
Educational Status:	
Illiterate	N=1, 1.4%
Read/Write	N=0, 0%
Primary School	N=3, 4.2%
Middle School	N=3, 4.2%
High School	N=26, 36.1%
Diploma	N=10, 13.9%
Bachelor’s Degree	N=26, 36.1%
Master’s Degree	N=2, 2.8%
PhD	N=1, 1.4%
Socioeconomic Status: Household Income Monthly:	
Less Than 5,000 SAR per Month	N=20, 27.8%
Between 5,000 and 20,000 SAR per Month	N=48, 66.7%
More Than 20,000 SAR per Month	N=4, 5.5%
Marital Status:	
Single	N=35, 48.6%
Married	N=34, 47.2%
Widowed	N=1, 1.4%
Divorced	N=2, 2.8%

Domain 2: psychological impact of anterior teeth caries

A one-way analysis of variance (ANOVA) was done, and we found that there is significant evidence of an overall difference in PIDAQ scores between groups (p-value= 0.01). Participants who perceived the presence of anterior caries had worse overall scores when compared to those who did not perceive having anterior caries (mean score= 43.52, 23.10, respectively). 

Similar patterns were seen in the dental self-confidence and social impact domains (p-value= 0.01, 0.02, respectively).

Tukey’s post hoc procedure found that patients who answered yes to the self-perception of anterior teeth caries question had worse average scores than those who answered no (p-value <0.05). The results of distribution of the PIDAQ and its components across participants grouped according to their answer to the self-perception of anterior teeth caries question are shown in Table 2 and Table 3. 

**Table 2:** The distribution of the PIDAQ and its components across participants grouped according to their answer to the self-perception of anterior teeth caries question *ANOVA test: p-value>0.05. **Tukey's post hoc: P-value >0.05 (identify specific between-group differences). PIDAQ: Psychosocial Impact of the Dental Aesthetics Questionnaire

Do You Have Decayed Anterior Teeth?	Yes (N=52)		No (N=10)		I don’t know (N=10)		p-value
	Mean	SD	Mean	SD	Mean	SD	
Dental Self-confidence	7.25**	6.43	13.60**	6.77	11.80	4.87	0.01*
Social Impact	12.13**	9.39	4.10**	6.61	7.10	9.17	0.02*
Psychological Impact	10.00	6.75	5.10	6.33	7.80	5.16	0.08
Esthetic Concern	4.63	4.26	3.50	4.65	1.70	2.67	0.12
PIDAQ Total Score	43.52**	21.51	23.10**	22.26	28.80	19.46	0.01*

**Table 3:** Patients’ self-perception of anterior teeth caries

Patients’ Self-perception of Anterior Teeth Caries Questions	
Do You Think You Need to See a Dentist Now or in the Next Couple of Weeks?	Yes (N=64, 88.9%)
	No (N=1, 1.4%)
	I don’t know (N=7, 9.7%)
Do You Have Decayed Anterior Teeth?	Yes (N=52, 72.2%)
	No (N=10, 13.8%)
	I Don’t Know (N=10, 13.9%)
At Present, Do You Think You Are in Need of Any Restorative Dental Treatment?	Yes (N=61, 84.7%)
	No (N=5, 7%)
	I Don’t Know (N=6, 8.3%)
Who Was the First One to Notice Your Anterior Teeth Caries?	Me (N=52, 72.2%)
	My Friends (N=1, 1.4%)
	My Family (N=5, 7%)
	My Dentist (N=14, 19.4%)

Domain 3: restorative treatment needs assessment

A two-sample T-test was done, and we found that patients with anterior teeth that required direct restoration had better dental self-confidence than those needing indirect restoration. The mean score was 9.59 and 5.60, respectively. This difference was statistically significant (p-value= 0.039).

Regarding social impact, psychological impact, and esthetic concerns, patients who needed indirect restorations had worse outcomes with higher average scores than those who needed direct restorations. However, these differences were not statistically significant.

On average, the overall PIDAQ score shows a more negative psychosocial impact of dental aesthetics on those needing indirect restoration than those needing direct restorations (mean score = 46.27, 36.63, respectively). However, the difference was not statistically significant (p-value = 0.14). The results of distribution of the PIDAQ and its components across participants grouped according to the restorative treatment needed are shown in Table 4.

**Table 4:** The distribution of the PIDAQ and its components across participants grouped according to the restorative treatment needed *Two-sample T-test: P-value>0.05. PIDAQ: Psychosocial Impact of the Dental Aesthetics Questionnaire

	Direct Restoration (N=57)		Indirect Restoration (N=15)		p-value
	Mean	SD	Mean	SD	
Dental Self-confidence	9.59	6.81	5.60	5.32	0.039*
Social Impact	9.49	9.21	13.47	9.88	0.15
Psychological Impact	8.74	6.20	10.07	8.32	0.50
Esthetic Concern	4.00	4.26	4.33	4.19	0.79
PIDAQ Total Score	36.63	21.90	46.27	24.09	0.14

## Discussion

Discolored teeth can significantly impact a person's well-being, as our society often associates a bright smile with beauty and confidence. To ensure successful dental treatment, addressing both patient satisfaction and aesthetic concerns is crucial [1,18]. The present study assessed the psychological impact of anterior dental caries and assessed the restorative treatment needs. The study's results revealed that participants who perceived the presence of anterior caries had significantly worse overall scores in terms of psychological impact than those who did not perceive having anterior caries. This indicates that individuals aware of their caries-affected anterior teeth experienced more negative psychological effects related to their dental aesthetics. The analysis of the PIDAQ scores revealed a noteworthy finding. Participants who required indirect restorations for their anterior teeth showed significantly higher overall PIDAQ scores, indicating a more negative psychosocial impact of dental aesthetics than those requiring direct restorations.

The dental self-confidence first factor pertaining to the PIDAQ indicated a notable influence of dental aesthetics on an individual's emotional state [16].

The study showed that the effects of psychological and social impact factors for those who perceive the presence of anterior caries had the highest score compared to those who don’t perceive having anterior dental caries; these individuals experienced the most significant emotional and social downsides. Studies have found that people with less attractive smiles and those who haven't had aesthetic dental treatment tend to experience a greater negative impact on their psychological and social well-being due to their teeth [2,19].

The fourth factor of the PIDAQ, called "esthetic," focuses on statements about individuals' dissatisfaction with their dental appearance when encountering mirror, photographic, and/or video images. Studies have established a connection between these findings and the primary motivation for orthodontic treatment: the desire to improve dental aesthetics [16].

In our study, a significant finding concerned patients' confidence levels who were having anterior teeth restored. Dental self-confidence was higher in those who needed direct restoration than in those who needed indirect restoration. Additionally, an analysis of the social implications, psychological effects, and aesthetic factors showed individuals who required indirect restorations generally expressed more severe concerns, as evidenced by higher average scores. Interestingly, the overall PIDAQ scores also showed a tendency towards a more negative psychosocial impact among patients needing indirect restoration compared to those needing direct restoration. This suggests that patients requiring indirect restorations may experience a greater psychosocial burden related to their dental aesthetics, potentially influenced by factors such as treatment complexity, duration, and perceived outcomes. Furthermore, prior research has demonstrated that teeth' alignment, color, and shape often constitute key factors leading to dissatisfaction with one's smile. When individuals are content with the alignment and shape of their teeth, they typically report enhanced emotional well-being and feelings of security [18].

This study investigates the relationship between anterior dental caries and the psychological impact on the affected individuals. Additionally, it explores potential treatment options for these individuals. Due to time constraints and internship requirements, the study was confined to patients at King Saud University clinics only, limiting the findings' generalizability. The study’s limitation is that the sample size was small, and the majority of participants were female. This is likely because emergency clinics tend to see more female than male patients.

To obtain a comprehensive understanding of the psychological impact of anterior dental caries and the associated treatment needs, it is essential to conduct further research. Ideally, such studies should encompass a more extensive and more diverse sample population, allowing for a broader representation of individuals with varying backgrounds.

Additionally, employing a longitudinal design in future research would be advantageous. This would involve studying participants over an extended period to track the psychological impact of anterior dental caries and treatment needs over time. A longitudinal approach would provide insights into the long-term effects, potential changes in psychological well-being, and the stability of treatment requirements over an extended period. It would enhance our understanding of the dynamic nature of the psychological impact and treatment needs associated with anterior dental caries.

## Conclusions

Caries of the anterior teeth significantly impacts individuals' psychological well-being, particularly in emotional and social aspects. Those perceiving the presence of anterior caries experience greater emotional distress and social implications. The need for restorative treatment, especially indirect restoration, is associated with higher psychosocial impact and lower dental self-confidence. Addressing the psychological effects of anterior dental caries and providing comprehensive restorative interventions are crucial for improving patients' well-being. Further research is needed to develop effective strategies to minimize psychosocial impact and enhance overall quality of life.

## Appendices

English version of the questionnaire

Domain 1: Demographic Data

Gender: (Female, Male).

Age: (18-23, 24-29, 30-35, 36-41, 42-47, Above 47).

Educational Status: (Illiterate, read/write, Primary School, Middle School, High School, Bachelor’s Degree, Master’s Degree, PhD).

Socioeconomic Status, Household Income (monthly): (Less than 5,000, Between 5,000-20,000, More than 20,000).

Marital Status: (Single, Married, Widowed, Divorced).

Domain 2: Psychological Impact of Anterior Teeth Caries 

Psychosocial Impact of Dental Aesthetics Questionnaire (PIDAQ) 

 0=not at all; 1=a little; 2=somewhat; 3=strongly; and 4=very strongly

Dental Self-Confidence

-I am proud of my teeth.

-I like to show my teeth when I smile.

-I am pleased when I see my teeth in the mirror.

-My teeth are attractive to others.

-I am satisfied with the appearance of my teeth.

-I find my tooth position to be very nice.

Social Impact

-I hold myself back when I smile so my teeth don't show so much.

-If I don't know people well I am sometimes concerned what they might think about my teeth.

-I'm afraid other people could make offensive remarks about my teeth.

-I am somewhat inhibited in social contacts because of my teeth.

-I sometimes catch myself holding my hand in front of my mouth to hide my teeth.

-Sometimes I think people are staring at my teeth.

-Remarks about my teeth irritate me even when they are meant jokingly.

-I sometimes worry about what members of the opposite sex think about my teeth.

Psychological Impact

-I envy the nice teeth of other people.

-I am somewhat distressed when I see other people's teeth.

-Sometimes I am somewhat unhappy about the appearance of my teeth.

-I think most people I know have nicer teeth than I do.

-I feel bad when I think about what my teeth look like.

-I wish my teeth looked better.

Aesthetic Concern

-I don't like to see my teeth in the mirror.

-I don't like to see my teeth in photographs.

-I don't like to see my teeth when I look at a video of myself.

Domain 3: Restorative Treatment Needs Assessment 

Do you think you need to see a dentist now or in the next couple of weeks? (Yes, No, I don’t know)

Do you have decayed anterior teeth? (Yes, No, I don’t know)

At present, do you think you are in need of any restorative dental treatment? (Yes, No, I don’t know)

Who was the first one to notice your anterior teeth caries? (Me, My friends, My family, My dentist)

Perception of the dentist to the presence of anterior teeth caries? (Yes, No)

Response of the dentists to the perception of restorative needs? (Direct restoration, Indirect restoration)
